# 

**Published:** 1860-05

**Authors:** 


					﻿No. XII.	MAY—1860.	Vol. XV.
NEW YORK
MONTHLY REVIEW
OF
AND
'S
BUFFALO MEDICAL JOURNAL.
EDITED BY AUSTIN FLINT, Jr, M.D,
Prof, of Physiology and Microscopic Anatomy in the New York Medical College.
And J. II. DOUGLAS, M.D.
NEW YORK:
HALL, CLAYTON & CO, PRINTERS,
4 6 PINE STREET.
				

